# Proband-mediated interventions to increase disclosure of genetic risk in families with a BRCA or Lynch syndrome condition: a systematic review

**DOI:** 10.1038/s41431-022-01200-z

**Published:** 2022-10-17

**Authors:** Alison Luk Young, Aalya Imran, Michael J. Spoelma, Rachel Williams, Katherine M. Tucker, Jane Halliday, Laura E. Forrest, Claire E. Wakefield, Phyllis N. Butow

**Affiliations:** 1 School of Medicine and Public Health, The University of Newcastle, Callaghan, NSW Australia; 2 Division of Obstetrics and Gynaecology, Royal North Shore Hospital, Sydney, NSW Australia; 3 Discipline of Psychiatry and Mental Health, School of Clinical Medicine, University of New South Wales, Sydney, NSW Australia; 4 Prince of Wales Clinical School, Faculty of Medicine, University of New South Wales, Randwick, NSW Australia; 5 Prince of Wales Hereditary Cancer Centre, Prince of Wales Hospital, Randwick, NSW Australia; 6 Murdoch Children’s Research Institute, Parkville, VIC Australia; 7 Department of Paediatrics, University of Melbourne, Parkville, VIC Australia; 8 Parkville Familial Cancer Centre, Peter MacCallum Cancer Centre, Melbourne, VIC Australia; 9 Sir Peter MacCallum Department of Oncology, The University of Melbourne, Melbourne, VIC Australia; 10 School of Women’s and Children’s Health, Faculty of Medicine and Health, UNSW, Sydney, NSW Australia; 11 Behavioral Sciences Unit, Kids Cancer Centre, Sydney Children’s Hospital, Randwick, NSW Australia; 12 School of Psychology, The University of Sydney, Sydney, NSW Australia

**Keywords:** Genetic counselling, Preventive medicine

## Abstract

Interventions to assist family communication about inherited cancer risk have the potential to improve family cancer outcomes. This review aimed to evaluate the efficacy of proband-mediated interventions employed within genetics clinics to increase disclosure of genetic risk to at-risk relatives. MEDLINE, Embase, CINAHL, PubMed and PsycINFO were searched for publications between 1990–2020. The quality of studies was assessed. From 5605 records reviewed, 9 studies (4 randomised control trials and 5 cohort studies) were included involving families with *BRCA1, BRCA2* and Lynch syndrome. Intervention delivery modes included genetic counselling with additional telephone or in-person follow-up, letters, videos, and decision aids. The percentages of at-risk relatives *informed* by the proband about their risk ranged from 54.0% to 95.5% in the intervention or family-mediated comparison group. Of those who were informed, 24.4–60.0% *contacted a genetics clinic* and 22.8–76.2% *had genetic testing after they were counselled at a genetics clinic*. Significant differences between intervention and control group were reported on all three outcomes by one study, and with relatives contacting a genetics clinic by another study. The studies suggest but do not conclusively show, that tailored genetic counselling with additional follow-up can increase both the proportion of informed relatives and relatives who contact the genetics clinic. With the increase in germline testing, interventions are required to consider the family communication process and address post-disclosure variables (e.g., relative’s perceptions, emotional reactions) through engagement with probands and relatives to maximise the public health benefit of identifying inherited cancer risk in families.

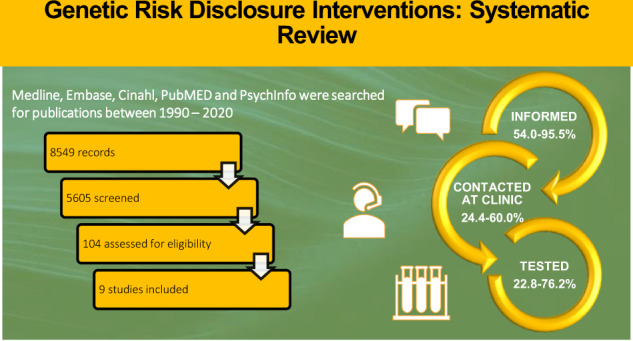

## Introduction

Rapid advances have been made in the identification of the aetiology of a wide range of inherited disorders, particularly regarding cancer predisposing germline pathogenic variants. Although variable, the heritable contribution to cancer overall has traditionally been estimated at 5–10%, however, more recent data indicate it may be as high as 33% (95% CI, 30–37%) due to common and rare variants [1]. The discovery of pathogenic variants has impacted clinical practice and outcomes by enabling provision of both personalised cancer risk estimates to individuals and recommendations for increased surveillance or risk-reduction strategies to improve survival.

The detection of pathogenic variants in asymptomatic individuals is potentially lifesaving. However, universal testing of all germline variants in the general population is not feasible and is cost-prohibitive, due to the overall low prevalence of pathogenic variants in the general population [2]. Thus, cascade testing, the direct testing of relatives of known pathogenic variant, is the primary approach employed by clinical genetic services [2]. Before testing of relatives can happen, disclosure of genetic results to the family must occur. This, however, can be problematic with dissemination of information often being left to the proband (the first individual in a family who receives genetic counselling and/or genetic testing), who is typically asked to inform their at-risk relatives about the availability of genetic testing. At-risk relatives are based on genetic relatedness as either first-degree (relative who shares 50% of their DNA with a particular individual; parents, offspring, and siblings) or second-degree (relative who shares 25% of their DNA; grandparent, grandchild, aunt/uncle, half-siblings, and niece/nephew) or third-degree relatives (relative who shares 12.5% of their DNA; first cousins).

Probands can encounter numerous challenges with disclosure. Common barriers include loss of contact with some family members, due to immigration, separation, death, or conflict [3]. Probands may also face emotional barriers such as guilt, anxiety, and concern about burdening relatives or a fear of relationship breakdown [3, 4]. Other barriers are related to lack of language skills, low levels of education, proband misunderstanding and lack of knowledge, or relying on other relatives for dissemination responsibilities [5]. Although reported disclosure rates vary from moderate to high [6–10], subsequent genetic testing in informed relatives remains suboptimal. In a study of dissemination within families with a known breast cancer pathogenic variant (*BRCA1* or *BRCA2*), the overall disclosure rate was 90%, but the rate of subsequent testing was 57% amongst relatives [11]. Some characteristics of relatives with lower BRCA testing rates include, younger relatives, male relatives, relatives of the paternal lineage, including second- or third-degree relatives [11].

Aiding probands with information and education about the risk for younger, male, and distant at-risk relatives may address some of the barriers.

Direct-mediated approaches (DMA), in which genetics clinics make direct contact with at-risk relatives, seem to be effective in disseminating information by overcoming the challenges faced by probands [12]. Yet, there remain privacy and confidentiality barriers preventing widespread implementation of DMA. Consequently, many genetic centres do not currently have legal authority to override a patient’s autonomy in dissemination of their genetic results [13] and/or guidelines do not mandate disclosure [14]. Furthermore, many countries do not have the legislation support to maintain a genetic registry which would be required to ensure DMA is conducted comprehensively and efficiently. Thus, the responsibility for family communication of genetic risk remains with probands. Given this situation it is important to synthesise the available data on proband-mediated interventions that address barriers and support probands to communicate with their relatives about genetic results, to better support them in this difficult task.

The aim of this paper was therefore to review the efficacy of proband-mediated interventions to increase disclosure of genetic risk to at-risk relatives, in relation to three main outcomes:

- the proportion of at-risk relatives informed about their risk
- the proportion of at-risk relatives contacting genetics clinics
- the proportion of at-risk relatives having genetic testing after they were counselled at a genetics clinic

## Materials and methods

### Database search procedure

Databases (MEDLINE, Embase, CINAHL, PubMed and PsycINFO) were searched using appropriate MeSH terms and keywords in the title and abstracts using the following terms: [intervention OR ((decision OR communication) AND (aid OR tool OR instrument))] AND family AND [cancer OR neoplasms OR genetics]. We exported the search results into EndNote X9 (Thomson Reuters) to be screened. The final search was conducted in November 2020, after which snowballing was conducted on all the included studies (both backwards via their reference lists, and forward through their citations as indexed by Google Scholar). The review followed the Preferred Reporting Items for Systematic Reviews and Meta-Analyses guidelines [15] (Supplementary file 1).

### Inclusion and exclusion criteria

Articles were included if they addressed the following:

1. *Family communication:* Communication between proband and at-risk relatives about the relative’s genetic risk of cancer and/or germline genetic testing.
2. *Intervention*: A clinically implementable intervention that had at least one component focused on improving family disclosure or educating probands about talking to relatives about hereditary cancer. Interventions included educational resources (decision aids, websites), and/or genetic counselling strategies.
3. *Study design*: Randomised control trials (RCTs), single-arm, and non-randomised control trials.
4. *Genetic condition*: hereditary breast-ovarian cancer (*BRCA1* or *BRCA2*) and hereditary non-polyposis colorectal cancer (HNPCC; Lynch syndrome)
5. *Outcomes*: Focused on three post-intervention outcomes: 1) the number of at-risk relatives informed about their risk, 2) the number of at-risk relatives contacting genetics clinics, and 3) the number of at-risk relatives having genetic testing after they were counselled at a genetics clinic
6. Published in a peer-reviewed journal between 1990 and 2020 to find relevant papers after DNA testing was introduced into clinical practice.

Articles were excluded if they:

1. Addressed general communication with spouses and extended relatives.
2. Did not address germline genetic testing.
3. Were a healthcare intervention that did not aim to assist probands in communicating with relatives.
4. Were conference proceedings, commentary, or reviews.
5. Were studies including interventions that aimed to improve family communication but only provided qualitative outcome data or provided total scores in which proband-specific data could not be extracted.

### Data extraction

**Fig. 1:**
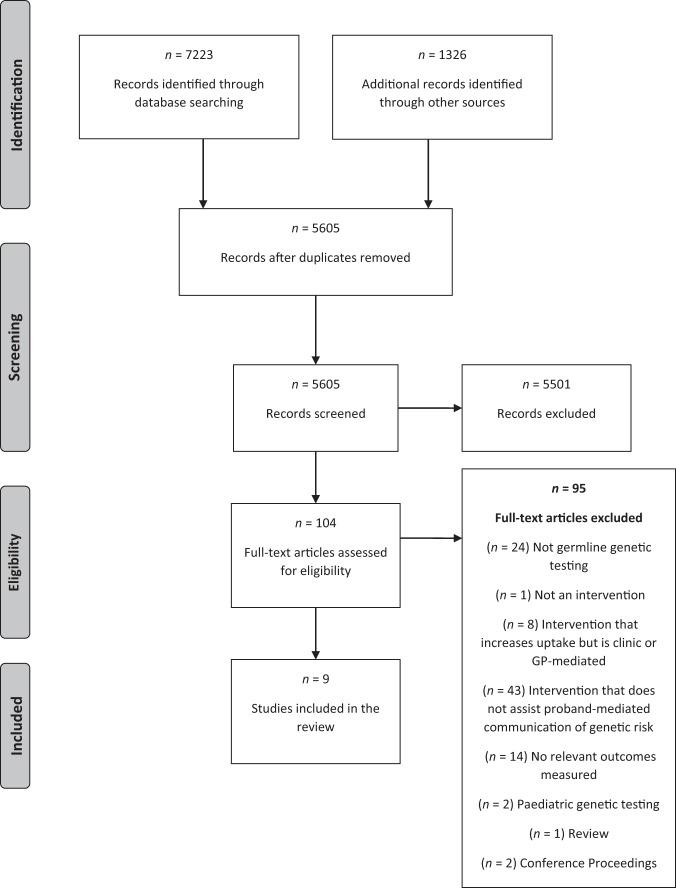
PRISMA flow chart. Identification – using the inclusion and exclusion criteria to retrieve articles from each database. Screening – The title/abstract for each article is screened and relevant articles are included. Eligibility – The full-text article is screened to assess their eligibility for inclusion. Inclusion – A final set of articles are included. ‘*n*’ refers to the number of articles.

We identified 5605 articles after duplicates were removed (Fig. 1). Two authors (A.L.Y. and M.J.S.) independently screened the titles and abstracts against the inclusion criteria (with an inter-rater agreement of 91%) and disagreements were resolved after discussion. A hundred and five full-text articles were reviewed, and a final nine articles met inclusion criteria. Two were identified through snowballing searches. Three reviewers (A.L.Y., M.J.S., A.I.) independently extracted data from these nine studies and cross-checked extractions to confirm their reliability. Study design, sample (s) characteristics (both intervention and control) was extracted including the proportion and statistical difference of at-risk relatives who were informed by a proband, contacted the genetics clinic, and had subsequent genetic testing. For the articles with multiple conditions, authors were contacted for additional data and analysis for families with *BRCA* or Lynch syndrome risk only. Study intervention characteristics were tabulated, and data was compared according to the three outcome categories in the review aim.

### Quality analysis

The Downs and Black [16] checklist for randomised and non-randomised healthcare intervention studies was used independently by two reviewers (A.L.Y., M.J.S.) to assess the quality of methodological reporting by studies. Discrepancies were resolved through discussion (Cohen’s Kappa = 0.91). Quality scores of each study are provided in Table 1 and were defined as: limited (<50%), adequate (50–70%), good (71–80%), or strong (score of >80%).

**Table 1:** Description of interventions and summary of outcomes.

Study (Country) (Citation)	Study Design	Condition	No. of probands/ families	Definition of at-risk relatives	Response rate	Intervention	Comparison/Control	Primary aim	Outcome	Quality Assessment category (%)
Atkan-Collan (Jyväskylä, Finland) [7]	Retrospective cohort study	HNPCC	110 families with known pathogenic variant	Family members at 50% risk of HNPCC	FMA: 90% DCA: 51%	Direct-contact approach (DCA) - clinic sent a direct letter to probands inviting them to the study, including subsequent genetic counselling and testing from the genetic clinic	Family-mediated approach (FMA) - proband informed relatives about genetic counselling and testing	1. Examine attitudes and reactions towards DCA vs FMA 2. Psychosocial responses	•76% (112/247) in the DCA and 86% (333/401) in the FMA study took the test and received results (*χ*^2^(1) = 3.58, *p* = 0.06) • 92% (103/113) approved of DCA by healthcare professionals • DCA group had a higher expectation of a positive future than FMA group (*F*(1.49) = 5.0, *p* = 0.03)	Adequate (63%)
Eijzenga (Amsterdam, Netherlands) [17]	RCT	*BRCA1*, *BRCA2*, HNPCC	763 probands (1^st^ in family to request counselling with >1 relative at risk)	1st, 2nd	336/763 (44%)	• Additional telephone counselling based on motivational interviewing provided by trained psychosocial workers. • Two-phases (1week and 4 months post), & follow-up questionnaires	No counselling	1. Knowledge of who to inform 2. Motivation and self-efficacy 3. Overall evaluation	• No between-group differences concerning knowledge of who to inform • Generally, participants had better knowledge of which first-degree relatives at risk needed to be notified (ICCs 0.75–0.91) as opposed to second-degree relatives (ICCs 0.06–0.72) • No difference in motivation • 96% (142/148) of participants found telephone counselling helpful	Good (78%)
Forrest (Victoria, Australia) [24]	Retrospective cohort study	Multiple: a balanced reciprocal chromosomal translocation, BRCA1,BRCA2, HNPCC, multiple endocrine neoplasia type 1, Peutz-Jegher syndrome, or an X-linked condition with reproductive implications	19 probands	1st, 2nd, 3rd	Not specified	Specific pedigree discussion, telephone follow-up calls 2–4 weeks post-result disclosure, review of family file and verification of whether at-risk relatives had made contact with the genetic service at 3-4 weeks post-result disclosure, recontacting index patient to document reasons, letter offered to be sent directly or via patient	Standard genetic counselling practices	1. The proportion of at-risk relatives who had made contact with the clinical genetics service within 2 years of the diagnosis of the index patient	• After 2-years, 61% (46/76) of intervention at- risk relatives definitely informed vs 36% (20/55) of control (*p* = 0.01) • Intervention group participants 2.6 times more likely to contact genetic clinic than control	Adequate (67%)
Garcia (Virginia, USA) [2]	Prospective nonrandomized pre- and post-intervention comparison pilot study	*BRCA1*, *BRCA2*	40 probands	1st	Not specified	Facing Our Risk of Cancer Empowered (FORCE) – written education materials provided prior to genetic counselling at oncology clinic visits	Pre-intervention standardised counselling	Determine whether incorporating materials as an adjunct to counselling is: 1. Acceptable to patients 2. Improve Knowledge of HBOC 3. Improve disclosure rates	• Majority found communication aid useful (90%; >4/5 on Likert scale) • Knowledge scores did not improve after use (*p* = 0.48) • No difference in disclosure rates between pre- and post-intervention cohorts (83% vs 77%, *p* = 0.26)	Limited (41%)
Hodgson (Victoria, Australia) [18]	RCT	Multiple: BRCA1,BRCA2, HNPCC, inherited cardiac conditions (e.g., long QT syndrome), X-linked conditions (Fragile X syndrome and Duchenne/Becker muscular dystrophy), autosomal recessive conditions (cystic fibrosis, deafness and spinal muscular atrophy)	95 probands	1st	95/167 (56.8%)	Specific genetic counselling using the Reciprocal Engagement Model (REM) and motivational interviewing via telephone at 3, 6 and 12 months from a trained genetic counsellor	Standard genetic counselling practices with no follow up contact	Determine whether the telephone counselling intervention increased frequency of contact with genetic services	• 25.6% (142/554) of the intervention group relatives contacted genetic services, compared with 20.9% (112/536) of the control group relatives (OR = 1.30; 95% CI = 0.70–2.42, *p* = 0.40) • 63.2% of intervention group high risk offspring made contact, compared with 6.7% of control offspring (OR = 24; 95% CI = 3.42–168.47, *p* = 0.001)	Good (74%)
Kardashian (California, USA) [25]	Pilot retrospective cohort study	*BRCA1*,*BRCA2*	37 probands	1st, 2nd, 3rd	19/37 (51.3%)	Sharing risk information tool (ShaRIT) is used in genetic clinic providing personalised recommendations, letter to family, written fact sheet/ education	Standard genetic counselling	1. Feasibility and acceptability of ShaRIT 2. Reported rates of sharing of results	• All ShaRIT group participants reported it was a useful resource • No difference in sharing rates between ShaRIT group (90%; *n* = 9) vs. control group (88%; *n* = 10; *p* = 1.00)	Adequate (56%)
Montgomery (Philadelphia, USA) [19]	RCT	*BRCA1*,*BRCA2*	345	1st	345/446 (77%)	Two sessions of a communication intervention involving the six-step breaking bad news model and education by a trained health educator	Two sessions of a wellness education intervention with general nutrition and exercise information by a trained health educator	1. Percentage of probands who shared genetic test results 2. Variables associated with sharing results	• No difference in sharing rates with all relatives between intervention group (54%; *n* = 74) vs. control (52.7%; *n* = 59; *p* = 0.83) • If the proband perceived that her relative was in favour of hearing her test result, she was more likely to share her test results (OR = 7.20, *p* < 0.0001; OR = 11.28, *p* < 0.0001, respectively) • Unambiguous results rather than inconclusive, female rather than male, children more than parents and daughters more than sons are likely to be informed	Strong (89%)
Roshanai (Uppsala, Sweden) [20]	RCT	*BRCA1*,*BRCA2*, HNPCC	210 probands	1st	147/210 (72%)	Additional genetic counselling session with a specialist nurse (provided after counselling), pedigree discussion, six-step breaking bad news model presented, written & video aids	Standard genetic counselling with a geneticist, no pedigree	Examine impact of intervention on: 1. Knowledge about genetics 2. Risk perception 3. Informed relatives 4. Satisfaction with counselling	• Increased level of knowledge was observed in the participants over time (*F* = 18 379, df = 2, *p* < 0.001), independent of the intervention • Increase in no. of probands who accurately estimated risk over time, significant difference between groups at 2 weeks • Majority of relatives reported receiving sufficient information, (75% intervention vs 67% control, but no difference) • Higher level of satisfaction in intervention group	Strong (81%)
Sermijin (Brussels, Belgium) [22]	Sequential prospective study	*BRCA1*,*BRCA2*	20 families	1st, 2nd, 3rd	89/172 (52%)	DCA: Direct-contact approach through letter and a 6-month follow-up phone call (Phase 2)	Family-mediated approach (FMA) with genetic counselling (family pedigree, discussion about barriers to communication, negative reactions, provided contact details of clinic, and 6-month visit to discuss informing relatives) (Phase 1)	1. Feasibility and safety of a stepwise interventional approach 2. Actively inform relatives	• Stepwise approach appeared feasible and nearly all participants that received a direct letter/phone had a positive experience • After DCA, 34% (42/125, 95% CI 26–42) of relatives were additionally reached, compared with the standard procedure	Adequate (59%)

*HNPCC* Hereditary nonpolyposis colorectal cancer or Lynch Syndrome, *BRCA* “BReast CAncer gene”—*BRCA1* and *BRCA2*, *FMA* family-mediated approach—the proband or family member informs their at-risk relative of their genetic risk, *DCA* Direct-contact approach—the genetic clinic makes direct contact with the at-risk relative apart from the proband, *1st* first degree relatives, *2nd* second degree relatives, *3rd* third degree relatives, *RCT* randomised control trial, *Index patient* an individual with the first known case of the pathogentic varinat in the family.

## Results

Of the nine studies included in this review (Fig. 1) there were four RCTs and five non-randomised studies. Seven studies exclusively evaluated interventions for probands with *BRCA* pathogenic variants, and one study evaluated an intervention for those with Lynch syndrome. The remaining two studies included *BRCA* and Lynch syndrome with additional genetic conditions, which were not included in the final analysis. Study characteristics and outcomes are summarised in Table 1. Results for the three primary outcome measures relevant to the research questions are provided in Tables 2–4 and are described below. The mean quality score [16] for the nine included papers was 65% (range = 41–89%). The findings and conclusions drawn from studies that had a quality score above 70% were considered to hold greater weight [17–20].

**Table 2:** Studies reporting about family members informing at-risk relatives about their genetic risk (Outcome 1).

Study	Type of study	Intervention and control group description	Intervention group			Comparison/Control group			Statistical significance
			No of at-risk relatives	Informed about their genetic risk	%	No of at-risk relatives	Informed about their genetic risk	%	
Eijzenga	RCT	Intervention: i) regular genetic counselling, included summary letter and testing at the department of Clinical Genetics ii) additional two-phase telephone counselling session based on motivational interviewing. Topics involved: a) Phase 1 identified at-risk relatives and what has been disclosed and a summary letter b) Phase 2 occurred if at-risk relatives were not informed according to the summary letter and included: correct information to provide appropriate relatives, building motivation, enhancing self-efficacy and brainstorming solutions to overcome barriers to disclosure Control: i) regular genetic counselling, included summary letter and testing at the department of Clinical Genetics	140	110^a^	78.5	150	114^a^	76.0	Not reported
Forrest	Retrospective cohort study	Intervention: i) reviewed pedigree ii) in-person specific discussion of pedigree to identify at-risk relatives and importance of disclosure iii) follow-up letter documenting importance of disclosure iv) 2–4 week post-result disclosure: telephone call with specific discussion about disclosure to at-risk relatives v) 3–6 months post-result disclosure: file review of whether at-risk relative have been contacted, if not, proband is recontact. Offer to provide letter explaining the genetic condition either distributed by proband or mailed directly to at-risk relative at the discretion of proband Control: i) reviewed pedigree ii) in-person general discussion of pedigree iii) follow-up letter documenting importance of disclosure iv) 2–4 week post-result disclosure: telephone call with general discussion about personal adjustment	60^c^	1st - 31 2nd - 7 3rd - 7 Total = 45^b,c^	75.0^c^	47^c^	1st - 6 2nd - 3 3rd - 7 Total = 16^b,c^	34.0^c^	Pearson chi^2^(1) = 18.0, *p* < 0.001^c^
Garcia	Prospective nonrandomized pre- and post-intervention comparison pilot study	Intervention: i) genetic counselling ii) supplementary written decision aid provided at the time of genetic testing - Facing Our Risk of Cancer Empowered (FORCE) resources: What you should know about genes and cancer brochure’, ‘Worksheet for sharing cancer information with the family’ and a ‘Family letter template’. Pre-intervention: i) genetic counselling	Not reported	Not reported	1^st^ – 83 (average)	Not reported	Not reported	1^st^ – 77 (average)	*P* = 0.26
Kardashian	Pilot retrospective cohort study	Intervention: i) in-person post-result genetic counselling informing patients of their BRCA result and discussion of implications of result for individual and their relatives ii) personalised medical report and lab results report iii) family pedigree iv) general information and resources e.g., support groups v) (provided in-person) a 3-4 page personalised medical report reviewing genetic testing process and implications of the result vi) a letter to family member notifying him/her of the pathogenic variant identified in relative vii) Fact-sheet FAQ for family members addressing cancer risk, cost of testing, insurance issues viii) contact information for genetic counsellors) nearest to eligible family members Control: i) in-person post-result genetic counselling informing patients of their BRCA result and discussion of implications of result for individual and their relatives ii) personalised medical report and lab results report iii) family pedigree iv) general information and resources e.g., support groups v) (mailed) a 3-4 page personalised medical report reviewing genetic testing process and implications of the result vi) (optional) a letter to family member notifying him/her of the pathogenic variant identified in relative	1st – 22; 2nd – 24; cousins – 27; Total = 73	1st – 20; 2nd – 18; cousins – 17; Total = 55	1st – 90.9; 2nd – 75.0; cousins – 63.0; Total = 75.3	1st – 36; 2nd – 32; cousin – 64; Total = 134	1st – 32^d^; 2nd – 12^d^; cousin – 25^d^; Total = 69^d^	1st – 88.9; 2nd – 37.5; cousins – 39.1; Total = 51.5^d^	1st – *p* = 1.00 2nd – *p* = 0.32 cousins – *p* = 0.86
Montgomery	RCT	Intervention: i) individual counselling involving the six-step breaking bad news model and education Topics included: a) identifying at-risk relatives b) choosing the communication format e.g., phone, letter c) assessing what relatives know and how much they might want to know At post-result disclosure: d) sharing test results with family members e) responding to family members’ emotional reaction to the disclosure f) providing genetic counselling resources for family members Control: i) a wellness education intervention before and after the disclosure of results Topics included: a) general nutritional information, the role of antioxidants and dietary supplements b) exercise information c) alcohol use and smoking cessation information	137 probands	74^e^	1st - 54.0 Total = 99.3	112 probands	110^e^	1st - 52.7 Total = 98.2	*P* = 0.83
Roshanai	RCT	Intervention: i) genetic counselling with Geneticist Topics include: a) education e.g., the differences between sporadic vs hereditary cancer, basic gentics, cancer risk b) provided estimates for at-risk relatives’ risk c) supplies information about genetic tesitng, surveilance programs, and explains the importance of cummunicating this information with relatives d) offered further contact if additional support is requested Additional session with specialist nurse directly after the counselling include: ii) explained the pedigree again, identifying at-risk relatives iii) asked about their intentions to inform at-risk relatives and brainstorm ways to overcome barriers iv) explained Buckman’s model of “Breaking Bad News” following the 6-steps as an aid to disclosure to at-risk relatives v) pamphlet about basic genetic concepts and specific information about their type of hereditary cancer vi) (shortly after the session) videotape from the counselling session, a copy of their medical records and a copy of the pedigree, to use in communicating the given information to their at-risk relatives Control: i) Genetic counselling with Geneticist Topics include: a) education e.g., the differences between sporadic vs hereditary cancer, basic genetics, cancer risk b) provided estimates for at-risk relatives’ risk c) supplies information about genetic testing, surveillance programs, and explains the importance of communicating this information with relatives d) offered further contact if additional support is requested Additional session with specialist nurse directly after the counselling included: ii) asked about their intentions to inform at-risk relatives iii) (mailed 8-months later) videotape from the counselling session	Not reported	Not reported	95.5 at 8-months^f^	Not reported	Not reported	89.0 at 8-months^f^	Not reported

*RCT* randomised control trial, *1st* first degree relatives, *2nd* second degree relatives, *3rd* third degree relatives

^a^Proband self-reported: The number of probands who shared information with at risk relatives based on a systematic discussion during an appointment with a psychosocial worker, who also identified whether correct informed was disclosed by using the summary letter as a gold standard.

^b^Proband self-reported and definitely informed: The number of probands who shared information with an at-risk relative as reported verbally to the genetic counsellor (intervention *n* = 9; control *n* = 2) and the number of at-risk relatives that were definitely informed if they had made personal contact with the genetic clinic or an interstate genetic service (intervention *n* = 36; control *n* = 14).

^c^Author re-analysed results to only include BRCA and Lynch syndrome.

^d^Calculated from published data.

^e^Proband self-reported: The number of probands sharing genetic test results with all first-degree relatives based on a 3-month follow-up survey.

^f^Relative self-reported: The number of relatives reporting that a proband had informed them about the proband’s own genetic counselling and about the content of given information.

**Table 3:** Studies reporting at-risk relatives contact with a genetic clinic after study intervention (Outcome 2).

Study	Type of Study	Intervention and control group description	Intervention			Comparison/Control			Statistical significance
			No of at-risk relatives	Made contact genetic clinic	%	No of at-risk relatives	Made contact genetic clinic	%	
Forrest^a^	Retrospective cohort study	Intervention: i) reviewed pedigree ii) in-person specific discussion of pedigree to identify at-risk relatives and importance of disclosure iii) follow-up letter documenting importance of disclosure iv) 2–4 week post-result disclosure: telephone call with specific discussion about disclosure to at-risk relatives v) 3–6 months post-result disclosure: file review of whether at-risk relative have been contacted, if not, proband is recontact. Offer to provide letter explaining the genetic condition either distributed by proband or mailed directly to at-risk relative at the discretion of proband Control: i) reviewed pedigree ii) in-person general discussion of pedigree iii) follow-up letter documenting importance of disclosure iv) 2–4 week post-result disclosure: telephone call with general discussion about personal adjustment	60	1st - 27 2nd - 5 3rd - 4 Total = 36	60.0	47	1st - 6 2nd - 3 3rd - 5 Total = 14	29.8	Pearson chi2(1)=9.7, *p* = 0.002
Hodgson^a^	RCT	Intervention: i) three-generation pedigree obtained at first clinic visit ii) genetic counselling iii) specific telephone genetic counselling aimed to enhance the ability of everyone to identify and overcome existing barriers in communicating with relatives iv) follow-up contact at three time-points (3, 6, 12-month) after diagnosis (number of calls tailored to everyone’s circumstance e.g., once all designated relatives were approached, calls were not required) Control: i) three-generation pedigree obtained at first clinic visit ii) genetic counselling	1st – 24; 2nd – 52; 3rd – 38; Total = 114	1st – 8; 2nd – 14; 3rd – 6; Total = 28	1st – 33.3; 2nd – 26.9; 3rd – 15.8; Total = 24.6	1st – 25; 2nd – 36; 3rd – 40; Total = 101	1st – 9; 2nd – 7; 3rd – 4; Total = 20	1st – 36.0; 2nd – 19.4; 3rd – 10.0; Total = 19.8	Chi sq=0.70, *p* *=* 0.40
Sermijin	Sequential prospective study	Intervention: Direct-contact approach (DCA; Phase 2) using the following: i) letter about familial cancer risk, predictive genetic testing, and optional genetic counselling, sent to at-risk relatives who had not yet come forward in first phase, or could not be contacted by the proband (proband anonymity is preserved) ii) 6-month follow-up phone call with relatives to have a final ascertainment of their wishes Comparison: Family-mediated approach (FMA; Phase 1) using the following: i) discussed in-detail the family pedigree and identifying at-risk relatives ii) discussion about barriers to communication and informed to disclose when the proband feels comfortable iii) provided contact details of clinic to distribute with relatives ii) 6-month second visit to discuss the status of informing relatives and difficulties encountered	172	47	27.3	**172**	**42**	**24.4**	Not reported; 34% (42/125, 95% CI 26–42) additionally reached with DCA

Bold relevant for the study.

*FMA* Family-mediated approach—the proband or family member informs their at-risk relative of their genetic risk, *DCA* Direct-contact approach—the genetic clinic makes direct contact with the at-risk relative apart from the proband, *RCT* Randomised control trial.

^a^Author re-analysed results to only include BRCA and Lynch syndrome.

**Table 4:** Studies reporting about at-risk relative’s genetic testing uptake after the intervention (Outcome 3).

Study	Type of Study	Intervention and control group description	Intervention			Comparison/Control			Statistical significance
			No. of at-risk relatives	Received genetic testing	%	No. of at-risk relatives	Received genetic testing	%	
Atkan-Collan	Retrospective cohort study	Intervention: Direct-contact approach (DCA) i) contact letter sent to high-risk subjects ii) pedigree iii) pre-test genetic counselling providing benefits and disadvantages of a predictive gene test were discussed, including psychological reactions and possible difficulties in employment or insurance coverage in the future iv) post-test counselling for subjects who had a positive predictive test v) referred for regular colonoscopies, and women were also referred for gynaecological examinations Comparison: Family-mediated approach (FMA) i) contact letter sent to high-risk subjects but content differs to the DCA according to assumed previous knowledge of cancer (two rounds of reminds were sent) ii) pedigree iii) pre-test genetic counselling providing benefits and disadvantages of a predictive gene test were discussed, including psychological reactions and possible difficulties in employment or insurance coverage in the future iv) post-test counselling for subjects who had a positive predictive test v) referred for regular colonoscopies, and women were also referred for gynaecological examinations	147	112	76.2	**401**	**333**	**83.0**	*X*^2^ = 3.58, *p* = 0.06
Forrest	Retrospective cohort study	Intervention: i) reviewed pedigree ii) in-person specific discussion of pedigree to identify at-risk relatives and importance of disclosure iii) follow-up letter documenting importance of disclosure iv) 2–4 week post-result disclosure: telephone call with specific discussion about disclosure to at-risk relatives v) 3–6 months post-result disclosure: file review of whether at-risk relative have been contacted, if not, proband is recontact. Offer to provide letter explaining the genetic condition either distributed by proband or mailed directly to at-risk relative at the discretion of proband Control: i) reviewed pedigree ii) in-person general discussion of pedigree iii) follow-up letter documenting importance of disclosure iv) 2–4 week post-result disclosure: telephone call with general discussion about personal adjustment	60^a^	1st - 24 2nd - 5 3rd - 3 Total = 32^a^	53.3^a^	47^a^	1st - 5 2nd - 3 3rd - 4 Total = 12^a^	25.5^a^	Pearson chi^2^(1) = 18.0, *p* < 0.001^a^
Garcia	Prospective nonrandomized pre- and post-intervention comparison pilot study	Intervention: i) genetic counselling ii) supplementary written decision aid provided at the time of genetic testing - Facing Our Risk of Cancer Empowered (FORCE) resources: What you should know about genes and cancer brochure’, ‘Worksheet for sharing cancer information with the family’ and a ‘Family letter template’ Pre-intervention: i) genetic counselling	22	1	4.5	6	0	0	Not provided
Hodgson	RCT	Intervention: i) three-generation pedigree obtained at first clinic visit ii) genetic counselling iii) specific telephone genetic counselling aimed to enhance the ability of each individual to identify and overcome existing barriers in communicating with relatives iv) follow-up contact at three time-points (3, 6, 12-month) after diagnosis (number of calls tailored to each individual’s circumstance e.g., once all designated relatives were approached, calls were not required) Control: i) three-generation pedigree obtained at first clinic visit ii) genetic counselling	1st – 24; 2nd – 52; 3rd – 38; Total = 114^a^	1st – 8; 2nd – 12; 3rd – 6; Total = 26a	1st – 33.3; 2nd – 23.1; 3rd – 15.8; Total = 22.8^a^	1st – 25; 2nd – 36; 3rd – 40; Total = 101^a^	1st – 8; 2nd – 6; 3rd – 3; Total = 17^a^	1st – 32.0; 2nd – 16.7; 3rd – 7.5; Total = 16.8^a^	chi sq = 1.19, *p* = 0.27^a^
Kardashian	Pilot retrospective cohort study	Intervention: i) in-person post-result genetic counselling informing patients of their BRCA result and discussion of implications of result for individual and their relatives ii) personalised medical report and lab results report iii) family pedigree iv) general information and resources e.g., support groups v) (provided in-person) a 3-4 page personalised medical report reviewing genetic testing process and implications of the result vi) a letter to family member notifying him/her of the pathogenic variant identified in relative vii) Fact-sheet FAQ for family members addressing cancer risk, cost of testing, insurance issues viii) contact information for genetic counsellors) nearest to eligible family members Control: i) in-person post-result genetic counselling informing patients of their BRCA result and discussion of implications of result for individual and their relatives ii) personalised medical report and lab results report iii) family pedigree iv) general information and resources e.g., support groups v) (mailed) a 3-4 page personalised medical report reviewing genetic testing process and implications of the result vi) (optional) a letter to family member notifying him/her of the pathogenic variant identified in relative	1st – 17; 2nd – 20; cousins – 27 Total = 65	1st – 3^b^; 2nd – 3^b^; cousins – 0; Total = 6^b^	1st – 17.6^b^; 2nd – 15.0^b^; cousins – 0 Total = 9.2^b^	1st – 36; 2nd – 33; cousins – 64 Total = 133	1st – 9; 2nd – 22; cousins – 0; Total = 32	1st – 25.0; 2nd – 66.7; cousins – 0; Total = 24.1	1st – *p* = 1.00 2nd – *p* = 0.10 cousins – *p* = N/A
Sermijin	Sequential prospective study	Intervention: Direct-contact approach (DCA; Phase 2) using the following: i) letter about familial cancer risk, predictive genetic testing, and optional genetic counselling, sent to at-risk relatives who had not yet come forward in first phase, or could not be contacted by the proband (proband anonymity is preserved) ii) 6-month follow-up phone call with relatives to have a final ascertainment of their wishes Comparison: Family-mediated approach (FMA; Phase 1) using the following: i) discussed in-detail the family pedigree and identifying at-risk relatives ii) discussion about barriers to communication and informed to disclose when the proband feels comfortable iii) provided contact details of clinic to distribute with relatives ii) 6-month second visit to discuss the status of informing relatives and difficulties encountered	47^c^	46	98	**42**^**d**^	**41**	**97.6**	Not reported

*Bold* relevant for the study.

FMA family-mediated approach—the proband or family member informs their at-risk relative of their genetic risk, *DCA* direct-contact approach—the genetic clinic makes direct contact with the at-risk relative apart from the proband, *1st* first degree relatives, *2nd* second degree relatives, *3rd* third degree relatives.

^a^Author re-analysed results to only include BRCA and Lynch syndrome.

^b^Calculated from published data.

^c^The 47/89 relatives came forward for predictive genetic counselling (53%; 95% CI 43–63).

^d^The 42/89 relatives came forward for predictive genetic counselling (47%; 95 % CI 37–57).

### Intervention components

Most of the interventions described involved either one or two genetic counselling appointments [21, 22] or an appointment with additional telephone genetic counselling sessions [17, 18] or extended sessions post-disclosure [19]. Specific communication techniques included motivational interviewing [17] and Robert Buckman’s [23] six-step ‘breaking bad news’ model [19, 20]. Two studies used a multiple-component intervention (face-to-face genetic counselling, written resources, and telephone support) [20, 24]. Two studies were written decision aids, either using Facing Our Risk of Cancer Empowered (FORCE) resources or using the Sharing Risk Information Tool [2, 25]. Two studies compared direct-contact by the genetics clinic with at-risk relatives (DCA) to the family-mediated approach (FMA; proband initiated contact) [21, 22]. For the purposes of this review, the FMA data were the focus of the intervention components and outcomes reported.

### Outcome 1: Number of at-risk relatives informed about their risk

Six studies provided a measure of the number of relatives who were informed post-intervention (Table 2). The intervention in four studies included additional/or enhanced genetic counselling [17, 19, 20, 24] and two studies evaluated a written decision aid [2, 25]. Five studies had a standard genetic counselling control [2, 19, 20, 24, 25] and one had no counselling as a control group [17]. Reported percentages of informed relatives in the intervention group ranged from 54.0–95.5%. One study [24] reported a significant intervention effect. Forrest et al. [24] found a greater percentage of at-risk relatives were informed when probands were provided with enhanced genetic counselling (specific pedigree discussion, telephone calls to index patient 2–4 weeks post-result disclosure providing guidance about how to approach relatives, and offer to distribute letter to relative directly or via index patient) to encourage family communication about *BRCA* and/or Lynch syndrome risk, compared to the control group (75% versus 34%, respectively, Pearson *χ*^2^(1) = 18.0, *p* < 0.001).

### Outcome 2: Number of at-risk relatives contacting genetics clinics

Three studies reported on the number of at-risk relatives contacting a genetics clinic post-intervention (Table 3). The three interventions varied, including two genetics clinic appointments focused on disclosure [22], counselling with three additional telephone genetic counselling sessions [18] or a multiple-component intervention [24]. Reported percentages of relatives in the intervention groups who contacted a genetics clinic ranged from 24.4% to 60.0%. One study had significant results [24]. Forrest et al. [24] reported a greater proportion of at-risk relatives contacting the clinic for *BRCA* or Lynch syndrome risk in the intervention group compared to the control group at 24-months post-intervention (60.0% versus 29.8%, respectively; Pearson *χ*^2^(1) = 9.7, *p* = 0.002).

### Outcome 3: Number of at-risk relatives having genetic testing after genetic counselling

Six studies provided a measure of genetic testing uptake post-intervention (Table 4).

Interventions included one or two genetic counselling appointments [21, 22], with additional telephone genetic counselling sessions [18], two written decision aids [2, 25], or a multi-component intervention [24]. The reported percentage of at-risk relatives who were counselled at a genetics clinic and had subsequent predictive genetic testing was between 22.8% and 76.2% (excluding outliers). Two studies were outliers. Sermijn et al. [22] had 53% (47/89) of relatives come forward for predictive genetic counselling when the process was proband-mediated, with 98% (46/47) of patients have predictive genetic testing. counselling. Garcia et al. [2] recruited patients at oncology outreach visits, provided written materials about family communication and although all 22 patients communicated with their relatives, only one relative had subsequent testing after 6-months (1/22; 4.5%). Forrest et al. [24] reported a greater testing rate for relatives in the intervention group compared to the control group, after excluding relatives who were referred interstate (53.3% versus 25.5%, Pearson *χ*^2^(1) = 18.0, *p* < 0.001).

## Discussion

The aim of this review was to examine the efficacy of proband-mediated interventions to improve disclosure of genetic risk to at-risk relatives. Three main outcomes of intervention efficacy were considered: the number of at-risk relatives informed, contacting genetics clinics, and having genetic testing after they were counselled at a genetics clinic. Of the nine studies that met the inclusion criteria, only four studies were of good quality [17–20] and only one study [24] reported a significant difference on all three outcome variables, with one additional study [18] reporting significant findings for attendance at genetics clinics. These findings highlight the need for more better-quality research measuring the outcomes of healthcare interventions to support proband communication to improve the awareness, communication, and uptake of genetic testing by at-risk relatives.

Four identified studies were RCTs that employed interventions delivering family communication focused genetic counselling by a healthcare professional (i.e., genetic counsellor, specialist nurse, or trained psychosocial workers). Two studies used the ‘breaking bad news’ [23] model [19, 20], either as a stand-alone intervention provided in the initial genetic counselling session [19] or at an additional visit with a nurse, supplemented by a suite of other resources (e.g., pamphlet, videotape, copy of medical records and pedigree) [20]. The other two studies provided telephone counselling, either as an adjunct to standard counselling study at three time-points (increased dosage) [18], or over two time-points to firstly explore what had been shared based on the patient’s summary letter, and secondly, to brainstorm solutions if barriers existed [17].

None of the RCTs reported a significant increase in disclosure and uptake of testing by at-risk relatives for *BRCA* and Lynch syndrome [18]. Baseline disclosure rates [2, 17] and motivation to disclose [19, 20, 24] was high in some studies, potentially influencing ability of interventions used in the included studies to add value. Another possibility is that intervention components were not sufficiently different to standard care, especially if the intervention involved an increased dosage of counselling [19], making it difficult to demonstrate differences.

The one retrospective cohort study by Forrest et al. [24], with significant results for all three outcomes, evaluated an intervention that was distinctly different to the counselling received by the control group. The intervention involved detailed, intentional genetic counselling involving pedigree review, documentation, and discussion of relatives not yet informed and discussion of communication barriers and potential solutions, as well as telephone follow-up at two time-points. Depending on the extent to which the proband had contacted at-risk relatives, a letter to relatives was optionally provided to the proband or directly to the relative at the final follow-up time-point. Although the participation rates were low, study results suggested that intentional counselling to improve the proportion of relatives informed and tested can be effective. However, given the small sample size of the study [24], further research is required to replicate findings.

Skills in conducting pedigree review and addressing family communication barriers are part of the repertoire of genetic counsellors and are easily applicable in a standard genetic counselling appointment [13]. Further research is needed to explore the feasibility of interventions with healthcare professionals other than genetic counsellors. Psychologists, nurses, social workers could also provide additional support to genetics clinics in equipping probands with the skills to communicate with their relatives, particularly as mainstreaming becomes more prevalent [26].

Forrest et al. [24] study also suggested that letters summarising important genetic results may be helpful, particularly if the proband lacks confidence in their ability to communicate these clearly and accurately. Importantly, a distinction needs to be made with regards to the studies that involved direct-contact via letter by the genetics clinic (DCA) in comparison to the family-mediated approach (FMA)as used in Forrest et al.. Studies comparing these two approaches [21] found no difference in psychological distress between DCA and FMA practices and greater uptake of genetic testing using a DCA protocol [22]. However, the DCA protocols used in these studies initially relied on the proband to contact relatives before sending a letter from the genetics clinic [21, 22], a process that mimics the protocol followed by FMA protocols [24]. Indeed, genetics clinics are required to follow the respective legislation and guidelines as to whether a purely DCA protocol is used [13, 14].

There are several limitations in identified studies that need to be addressed in future studies. First, more rigorous studies with adequate power to test hypotheses are required to determine best practice for improving disclosure rates, given that only four studies were RCTs. Second, consideration of a preferred primary outcome is required. The primary outcomes of some of the current studies included genetic risk knowledge, self-efficacy, risk perception and motivation. Although these are important process variables, it is important to measure objective outcomes such as the three outcome variables assessed in this review, using audit and survey data [24]. Third, control conditions (particularly those involving standard genetic counselling) need to be well described, to allow identification of key differences between the intervention and control group [2, 17, 24]. Fourth, given that some of the included studies reported high baseline disclosure rates, targeting recruitment to families facing difficulties with communication may increase the likelihood of detecting intervention effects. This gap in our understanding could uncover the areas where interventions are lacking, including but not limited to, consideration of the relative’s perceptions, assumptions, and experiences. From the family communication literature, it is known that a proband’s emotional reaction towards finding out their test results and their subsequent preventative decisions can sometimes cast a bleak picture of the future, one which their children do not wish to follow [3]. Sharing of testimonials and family group sessions can allow relatives to consider the experiences of others. Moreover, interventions could target certain at-risk groups of relatives known to have lower uptakes of testing, e.g., those in the parental lineage and male relatives. Knowledge of their risk could be increased through awareness groups like the The Movember Foundation (https://movember.com) and engagement with visual and social media platforms.

Limitations of the current review also need to be considered. Variability in the content delivered, specifically standard care procedures, the control/comparison group, frequency of follow-up, outcomes, and definitions of at-risk relatives in identified studies disallowed a quantitative synthesis of trial findings. The percentage of informed at-risk relatives is based on self-reports made by probands, which provides a proxy for the true amount informed, given that it is unethical to approach relatives directly. Moreover, the percentage of relatives informed is dependent on the percentage of probands informed. Studies not conducted in English were excluded, thus some relevant papers may have been omitted.

In summary, a limited number of interventions have measured the efficacy of interventions to improve disclosure of genetic risk to family members. Intentional genetic counselling practices, including pedigree review and strategies to improve communication, with additional follow-up, could improve disclosure rates for families with *BRCA* and Lynch syndrome. Yet the findings for this are minimal and there is no RCT that has shown a significant result. Given that many of the studies reported a high rate of disclosure but a low uptake of testing amongst relatives, future research should focus on examining post-disclosure variables (e.g., relative’s perceptions and understanding of the test result, emotional reactions towards’ their family member’s decisions) to determine whether these pose barriers to uptake of genetic testing at at-risk family members. The use of a process model, such as the one developed by Lafrenière et al. [27] provides a framework for understanding the process of communicating genetic test results to family members. Targeted interventions would benefit from drawing from qualitative research and using the model in the pre-intervention development stage, focusing not only on the content of the proband’s disclosure, but also the emotions or sentiments they convey, the decision-making process and reactions of the relatives. With the increase in genetic testing and the demand on genetics clinics to address family communication, this remains a critical area for further research.

## Supplementary information

Supplementary File 1. PRISMA checklist

## Data Availability

Data sharing is not applicable to this article as no new data were created or analysed in this study.

## Code availability

The data generated or analysed during this study can be found within the published article and its supplementary files, along with requests from the authors.

## References

[1] Mucci LA, Hjelmborg JB, Harris JR, Czene K, Havelick DJ, Scheike T (2016). Familial risk and heritability of cancer among twins in Nordic countries. JAMA.

[2] Garcia C, Sullivan MW, Lothamer H, Harrison KM, Chatfield L, Thomas MH (2020). Mechanisms to increase cascade testing in hereditary breast and ovarian cancer: Impact of introducing standardized communication aids into genetic counseling. J Obstet Gynaecol Res.

[3] Young AL, Butow PN, Rhodes P, Tucker KM, Williams R, Healey E (2019). Talking across generations: family communication about BRCA1 and BRCA2 genetic cancer risk. J Genet Couns.

[4] McGarragle KM, Hare C, Holter S, Facey DA, McShane K, Gallinger S (2019). Examining intrafamilial communication of colorectal cancer risk status to family members and kin responses to colonoscopy: a qualitative study. Hered Cancer Clin Pract.

[5] Healey E, Taylor N, Greening S, Wakefield CE, Warwick L, Williams R (2017). Quantifying family dissemination and identifying barriers to communication of risk information in Australian BRCA families. Genet Med.

[6] Cheung EL, Olson AD, Yu TM, Han PZ, Beattie MS (2010). Communication of BRCA results and family testing in 1103 high-risk women. Cancer Epidemiol Biomark Prev.

[7] Aktan-Collan KI, Kääriäinen HA, Kolttola EM, Pylvänäinen K, Järvinen HJ, Haukkala AH (2011). Sharing genetic risk with next generation: mutation-positive parents’ communication with their offspring in Lynch Syndrome. Fam Cancer.

[8] Pentz RD, Peterson SK, Watts B, Vernon SW, Lynch PM, Koehly LM (2005). Hereditary nonpolyposis colorectal cancer family members’ perceptions about the duty to inform and health professionals’ role in disseminating genetic information. Gen Test.

[9] Ishii N, Arai M, Koyama Y, Ueno M, Yamaguchi T, Kazuma K (2011). Factors affecting encouragement of relatives among families with Lynch syndrome to seek medical assessment. Fam Cancer.

[10] Ricker CN, Koff RB, Qu C, Culver J, Sturgeon D, Kingham KE (2018). Patient communication of cancer genetic test results in a diverse population. Transl Behav Med.

[11] Finlay E, Stopfer JE, Burlingame E, Evans KG, Nathanson KL, Weber BL (2008). Factors determining dissemination of results and uptake of genetic testing in families with known BRCA1/2 mutations. Gen Test.

[12] Menko FH, ter Stege JA, van der Kolk LE, Jeanson KN, Schats W, Moha DA (2018). The uptake of presymptomatic genetic testing in hereditary breast-ovarian cancer and Lynch syndrome: A systematic review of the literature and implications for clinical practice. Fam Cancer.

[13] Young AL, Butow PN, Tucker KM, Wakefield CE, Healey E, Williams R (2020). When to break the news and whose responsibility is it? A cross-sectional qualitative study of health professionals’ views regarding disclosure of BRCA genetic cancer risk. BMJ Open.

[14] Dheensa S, Fenwick A, Shkedi-Rafid S, Crawford G, Lucassen A (2016). Health-care professionals’ responsibility to patients’ relatives in genetic medicine: a systematic review and synthesis of empirical research. Genet Med.

[15] Page MJ, McKenzie JE, Bossuyt PM, Boutron I, Hoffmann TC, Mulrow CD, et al. The PRISMA 2020 statement: an updated guideline for reporting systematic reviews. BMJ. 2021;372:n71. 10.1136/bmj.n71 PMC8005924 33782057

[16] Downs SH, Black N (1998). The feasibility of creating a checklist for the assessment of the methodological quality both of randomised and non-randomised studies of health care interventions. J Epidemiol Community Health.

[17] Eijzenga W, de Geus E, Aalfs CM, Menko FH, Sijmons RH, de Haes HCJM (2018). How to support cancer genetics counselees in informing at-risk relatives? Lessons from a randomized controlled trial. Patient Educ Couns.

[18] Hodgson J, Metcalfe S, Gaff C, Donath S, Delatycki MB, Winship I (2016). Outcomes of a randomised controlled trial of a complex genetic counselling intervention to improve family communication. Eur J Hum Genet.

[19] Montgomery SV, Barsevick AM, Egleston BL, Bingler R, Ruth K, Miller SM (2013). Preparing individuals to communicate genetic test results to their relatives: report of a randomized control trial. Fam Cancer.

[20] Roshanai AH, Rosenquist R, Lampic C, Nordin K (2009). Does enhanced information at cancer genetic counseling improve counselees’ knowledge, risk perception, satisfaction and negotiation of information to at-risk relatives? A randomized study. Acta Oncol.

[21] Aktan-Collan K, Haukkala A, Pylvänäinen K, Järvinen HJ, Aaltonen LA, Peltomäki P (2007). Direct contact in inviting high-risk members of hereditary colon cancer families to genetic counselling and DNA testing. J Med Genet.

[22] Sermijn E, Delesie L, Deschepper E, Pauwels I, Bonduelle M, Teugels E (2016). The impact of an interventional counselling procedure in families with a BRCA1/2 gene mutation: efficacy and safety. Fam Cancer.

[23] Buckman R (2017). How to break bad news.

[24] Forrest LE, Burke J, Bacic S, Amor DJ (2008). Increased genetic counseling support improves communication of genetic information in families. Genet Med.

[25] Kardashian A, Fehniger J, Creasman J, Cheung E, Beattie MS (2012). A pilot study of the sharing risk information tool (ShaRIT) for families with hereditary breast and ovarian cancer syndrome. Hered Cancer Clin Pract.

[26] Patch C, Middleton A (2019). Point of view: an evolution from genetic counselling to genomic counselling. Eur J Med Genet.

[27] Lafrenière D, Bouchard K, Godard B, Simard J, Dorval M (2013). Family communication following BRCA1/2 genetic testing: a close look at the process. J Genet Couns.

